# A systematic review and meta-analysis comparing conservative and surgical treatments for acute patellar dislocation in children and adolescents

**DOI:** 10.1186/s43019-023-00189-z

**Published:** 2023-06-22

**Authors:** Dong-Yeong Lee, Dong-Geun Kang, Ho-Seung Jo, Se-Joon Heo, Ji-Ho Bae, Sun-Chul Hwang

**Affiliations:** 1 Department of Orthopaedic Surgery, Barun Hospital, Jinju, Republic of Korea; 2 Department of Orthopaedic Surgery, College of Medicine and Gyeongsang National University and Changwon Hospital, Changwon, Republic of Korea; 3 Department of Orthopaedic Surgery, SMG Yeonse Hospital, Changwon, Republic of Korea; 4 Department of Orthopaedic Surgery, College of Medicine and Gyeongsang National University and Hospital, Jinju, Republic of Korea

**Keywords:** Patellar dislocation, Patellar instability, Medial patellofemoral ligament, Reconstruction, Meta-analysis

## Abstract

**Purpose:**

This study sought to clarify treatment evidence to treat patellar dislocation by evaluating which treatment could yield better improvement of clinical outcomes for acute patellar dislocation in children and adolescents 18 years of age or younger.

**Materials and methods:**

MEDLINE, EMBASE, and the Cochrane Central Register of Controlled Trials electronic databases were searched for relevant articles comparing clinical outcomes of conservative and surgical treatments for acute patellar dislocation in children and adolescents published from March 2008 to August 2022. Data searching, extraction, analysis, and quality assessment were performed on the basis of the Cochrane Collaboration guidelines. The quality assessment of each study was investigated using the Physiotherapy Evidence Database (PEDro) critical appraisal scoring system and Newcastle–Ottawa Quality Assessment Scale scores. To calculate the overall combined effect size for each outcome, Review Manager Version 5.3 (The Cochrane Collaboration, Software Update, Oxford) was employed.

**Results:**

Three randomized controlled trials (RCTs) and one prospective study were investigated. In terms of pain [mean difference (MD) 6.59, 95% confidence interval (CI) 1.73–11.45, *I*^2^ 0%], there were significantly better outcomes in conservative group. Nevertheless, there were no significant differences in any evaluated outcomes such as redislocation [risk ratio (RR) 1.36, 95% CI 0.72–2.54, *I*^2^ 65%], Kujala score (MD 3.92, 95% CI −0.17 to 8.01, *I*^2^ 0%), Tegner score (MD 1.04, 95% CI −0.04 to 2.11, *I*^2^ 71%), or subjective results (RR 0.99, 95% CI 0.74–1.34, *I*^2^ 33%) between conservative and surgical treatment groups.

**Conclusions:**

Despite better pain outcomes with conservative group, the present study revealed no significant differences in clinical outcomes between conservative treatment and surgical treatment in children and adolescents with acute patellar dislocation. Since there are no significant differences in clinical outcomes between the two groups, routine surgical treatment is not advocated for treating acute patellar dislocation in children and adolescents.

## Introduction

Acute patellar dislocation is a common knee injury in children and adolescents, with an incidence of 0.3–1.2 per 1000 in children aged 9–15 years [1–3]. Without appropriate treatment, patellar dislocation can lead to subsequent redislocation, painful instability, anterior knee pain, and patellofemoral degeneration [4–6]. Thus, effective treatments are required.

Orthopedic surgeons face difficulties in treating patellar dislocation due to complexity of the procedures and unsatisfactory results, which often lead to frequent recurrence. Previously, conservative treatment was considered the preferred option. However, the recurrence rate was high (30–70%) in those without an operative treatment, with the highest rate observed in younger patients [2, 7, 8]. For these reasons, recently there has been a trend toward surgical treatment by medial patellofemoral ligament (MPFL) stabilization. Several studies have reported that surgery is superior to conservative treatment regarding redislocation rate, quality of life, and sporting function in children and adolescents [7, 9, 10]. However, a high incidence of degenerative changes in patellofemoral joint with influence on subjective knee function in long-term follow-ups of these patients has also been reported [11, 12].

Our group has previously published a study on primary patellar dislocation in adults and reported that conservative and surgical treatments have similar clinical results [13]. Furthermore, various systematic reviews and meta-analyses that examined the general population have found that surgical treatment results in fewer incidents of redislocation and better knee function than conservative treatment in the short term, while long-term outcomes are comparable [14–16]. However, children and adolescents have different anatomic characteristics due to their immature musculoskeletal growth. Hence, it would not be appropriate to adapt results of studies on adults to children and adolescents. In addition, children and adolescents have characteristics that indicate rapid growth. Their growth plate is open, making it difficult to choose the surgical option. Therefore, clinical results according to treatments for acute patellar dislocation in adults and children/adolescents should be analyzed separately.

Restoring the function of the medial patellofemoral ligament (MPFL) is crucial in the treatment of patellar dislocation. The MPFL serves as the primary restraint against lateral patellar translation and provides 50–60% of the medial restraining force against such translation [4, 17, 18]. The MPFL is often damaged during an acute patellar dislocation in skeletally immature patients, with the majority of injuries occurring at the site of the ligament’s attachment to the patella [19–21]. Adult patients with patellar dislocation typically exhibit a higher prevalence of femoral-based lesions than children, although the injury is usually multifocal and frequently involves the patellar attachment site [22–24]. For these reasons, skeletally immature children might have a different natural history after patellar dislocation than adult populations [25, 26]. In particular, in terms of treatment of patellar dislocation, conservative treatment of first time pediatric acute patellar dislocation has been associated with up to a 69% failure rate [25]. Thus, attention has shifted toward surgical treatment for addressing patellar instability [7, 10, 27]. While multiple studies have shown that surgical treatment results in fewer redislocations and improved knee function compared with conservative treatment in all patients in the short term, long-term outcomes tend to be similar [14–16]. In addition, how surgical treatment will affect children and adolescents for treating acute patellar dislocation remains a controversy.

Thus, the purpose of this review was to summarize treatment evidence to treat patellar dislocation by evaluating which treatment could yield better improvement in stability and functional recovery for acute patellar dislocation in children and adolescents. We hypothesized that surgical treatment might not provide better outcomes after acute patellar dislocation in children and adolescents.

## Materials and methods

### Study selection

The present study employed Cochrane Review methods and followed the Preferred Reporting Items for Systematic Reviews and Meta-Analyses (PRISMA) statement for reporting. Controlled vocabulary and free text words listed in the Appendix were utilized to search the MEDLINE, EMBASE, and Cochrane Central Register of Controlled Trials databases to identify relevant studies. We included all relevant studies, regardless of their publication type (e.g., article, poster, conference article, instructional course lectures), language, publication journal, or year of publication. The search was updated in August 2022, and reference lists of the identified studies and review articles were examined for any additional relevant publications. Moreover, we reviewed the reference lists of the investigated studies to identify any further publications that were not found through manual or electronic searches. We also checked for duplication in cases where the same author had two or more studies. If duplicated, only the latest study was included.

### Inclusion and exclusion criteria

Studies were included in our investigation if they met the following criteria: (1) studies on children and adolescent subjects (less than 18 years of age) who received treatment for acute patellar dislocation, (2) studies that compared clinical outcomes of conservative treatment and surgical treatment for acute patellar dislocation, and (3) those with level I or level II evidence. Exclusion criteria were: (1) studies on revision cases or old dislocation, (2) study subjects who had congenital abnormality or congenital disease, (3) studies that evaluated only adult subjects, (4) studies that only reported non-clinical outcome measures or intraoperative measures, (5) studies with level III, IV, or V evidence (retrospective studies, case report, technical note, or letters to the editor), (6) review articles, (7) biomechanical studies, and (8) in vitro studies.

### Data collection and analysis

Two authors independently reviewed the titles and abstracts of studies identified through the search strategy. Full papers were then evaluated for final inclusion. Any uncertainty regarding study inclusion was resolved through discussion and consensus. The authors independently extracted eligible data onto predefined forms and checked for accuracy. We gathered information on various study characteristics, including details about the authors, journal, study design, publication year, patient demographics such as sex and age, number of participants, treatment interventions (conservative methods and surgical techniques), and follow-up duration (Table 1). Treatment interventions used in this study were physical therapy (PT) or brace immobilization in the conservative treatment group, and in the surgical treatment group, MPFL repair or lateral retinacula release (LRR), or these two methods were used in combination. In addition, the clinical results of the included studies were evaluated, regarding redislocation, postoperative complications, Kujala score, Tegner score, pain, surgery after initial intervention, and subjective result (Table 2). “Pain” was evaluated on the basis of visual analogue scale (VAS) score [8] or pain score of knee injury and osteoarthritis outcome score (KOOS) [28]. In addition, “subjective results” were evaluated on the basis of medical record [8] or questionnaire in a telephone interview [10]. The data extracted from the eligible studies were used to calculate the number of participants or the mean and standard deviation (SD) of demographic and clinical outcome measures for each group, which were then reviewed and summarized in the results and tables.

**Table 1:** Characteristics of included studies

Study	Journal	Study design	Year	Sample Size	Age (years)	Sex (M:F)	Intervention	Follow-up (m)
Askenberger et al. [28]	American Journal of Sports Medicine	RCT	2018	Conservative: 37 Surgical: 37	13.0 ± 1.1 13.2 ± 1.1	17:20 19:18	Conservative: PT and knee brace that was lateral stabilizing Soft tissue brace for 4 weeks Surgical: arthroscopic MPFL repair, followed by a soft cast splint for 4 weeks	24
Apostolovic et al. [31]	International Orthopaedics	Prospective study	2011	Conservative: 23 Surgical: 14	14.3 ± 1.0 13.1 ± 1.0	4:19 5:9	Conservative: immobilization for 3 weeks, quadriceps exercise Surgical: MPFL repair or arthroscopic LRR	73.2
Regalado et al. [10]	Knee Surgery Sports Traumatology Arthroscopy	RCT	2016	Conservative: 15 Surgical: 15	13.5 (8–16) 13.5 (8–16)	9:11 5:11	Conservative: PT and knee brace for 4 weeks Surgical: Fulkerson type I, LRR Fulkerson type II, III, IV, proximal and distal LRR and medial imbrications	72
Palmu et al. [8]	Journal of Bone and Joint Surgery Am	RCT	2008	Conservative: 28 Surgical: 32	13.0 ± 2.0 13.0 ± 2.0	9:19 5:27	Conservative: immobilization with a removable knee extension orthosis or patella-stabilizing orthosis for 6 weeks Surgical: MPFL repair or MPFL repair + LRR, or only LRR	168

*RCT* randomized controlled trial, *PT* physical therapy, *MPFL* medial patellofemoral ligament, *LRR* lateral retinacula release

**Table 2:** Clinical outcomes of conservative and surgical treatments in included studies

Study	Group (*n*)	Redislocation	Postoperative complications	Kujala score	Tegner score	Pain	Surgery after initial intervention	*Subjective results
Askenberger et al. [28]	Conservative: 37 Surgical: 37	16 8	2	95.9 ± 7.2 90.9 ± 13.0	5.0 ± 1.4 4.5 ± 2.0	89.3 ± 11.7 83.1 ± 16.8	Conservative: 6 Surgical: 0	NP
Apostolovic et al. [31]	Conservative: 23 Surgical: 14	1 2	NP	NP	NP	NP	Conservative: 4 Surgical: 4	NP
Regalado et al. [10]	Conservative: 15 Surgical: 15	11 5	3	NP	NP	NP	Conservative: 4 Surgical: 0	Conservative: 11 Surgical: 13
Palmu et al. [8]	Conservative: 28 Surgical: 32	20 24	2	84.0 ± 13.0 83.0 ± 18.0	6.0 ± 1.9 4.4 ± 1.4	91.0 ± 10.0 84.0 ± 18.0	Conservative: 11 Surgical: 16	Conservative: 21 Surgical: 21

*VAS* visual analogue scale, *KOOS* Knee injury and Osteoarthritis Outcome Score, *QOL* quality of life, *NP* not provided

*Subjective result: scoring or number of patients with excellent or good subjective results were evaluated

### Assessment of methodological quality

Two investigators independently assessed methodological qualities of each study using the Physiotherapy Evidence Database (PEDro) critical appraisal scoring system, a reliable tool for assessing RCTs and Newcastle–Ottawa Quality Assessment Scale scores for non-RCTs [29, 30]. Any disagreement between authors was resolved through discussion or through a review by the third investigator. Publication bias was not evaluated due to a low statistical power as the number of included studies was less than ten in each field of research.

### Statistical analysis

We used Review Manager Version 5.3 (The Cochrane Collaboration, Software Update, Oxford) to calculate the overall pooled effect size for each outcome. For studies involving only randomized trials and prospective studies, we conducted a meta-analysis using a random effects model. For continuous outcomes, we used the inverse variance method to determine the mean difference (MD) with a 95% confidence interval (CI). For binary outcomes, we calculated the risk ratio (RR) between groups using the Mantel–Haenszel method. We evaluated statistical heterogeneity among studies using I-squared (I^2^), with values of 25%, 50%, and 75% considered low, moderate, and high, respectively, and Cochrane’s Q statistic (chi-squared test). A *p*-value < 0.10 was considered statistically significant for heterogeneity.

## Results

### Identification of studies

**Fig. 1:**
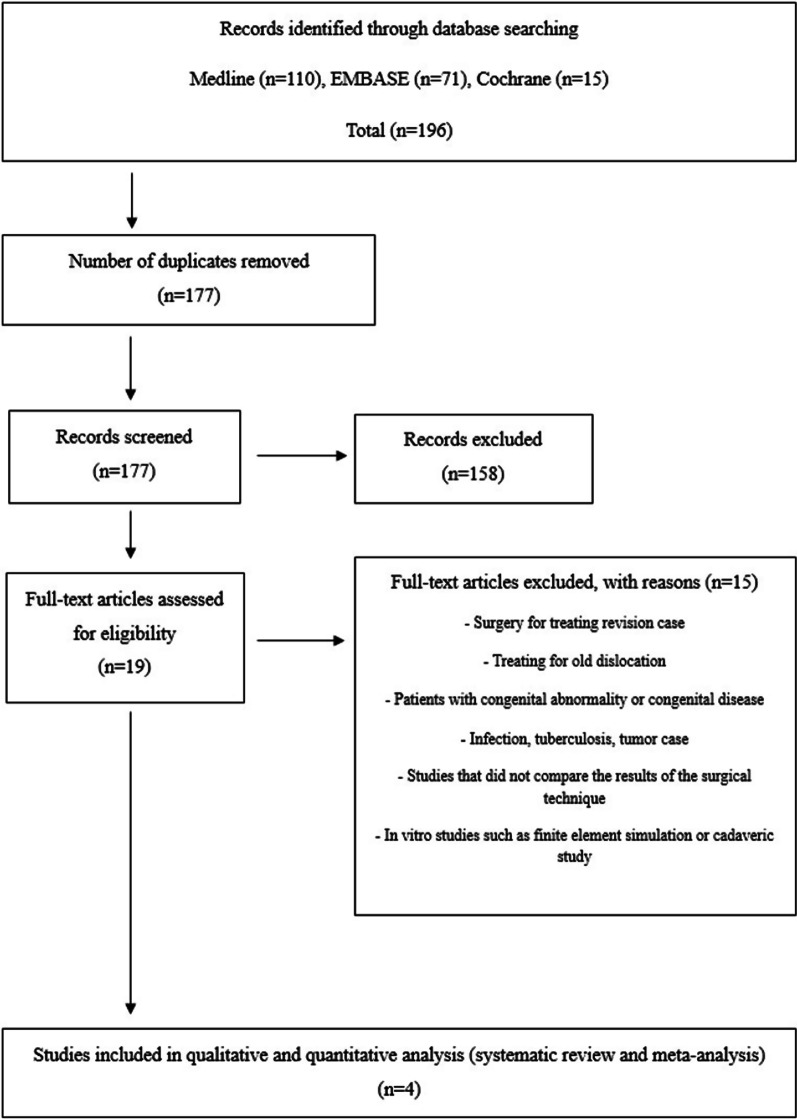
Preferred Reporting Items of Systematic Reviews and Meta-Analyses (PRISMA) flow diagram

At the start of this study, 196 articles were found to be potentially relevant. After eliminating 19 duplicates and screening the remaining 177 articles using titles and abstracts, 19 articles were deemed potentially relevant for full-text review. However, upon full-text review, 15 of these articles were excluded due to a lack of essential data. Ultimately, four clinical studies were included for data extraction and meta-analysis (Fig. 1) [8, 10, 28, 31].

### Quality of included studies

To evaluate the methodological quality, we used the PEDro critical appraisal scoring system for RCTs and Newcastle–Ottawa Quality Assessment Scale (maximum 9 points) scores for non-RCTs. The average PEDro score for the RCTs was 8.7 points, with a range of 8–9, indicating that most studies were of good or fair quality. All studies met the eligibility criteria and included randomization, adequate follow-up, intention-to-treat analysis, between-group analysis, and point estimates and variability. However, some studies were found to have used an inadequate blinding method, which could potentially have led to detection bias. Despite this weakness, the majority of the study designs scored over 8, indicating a low risk of bias. In addition, the non-RCT study had Newcastle–Ottawa Quality Assessment Scale scores ≥ 8 points, indicating a low risk of bias of included non-RCT studies.

### Clinical results of included studies

#### Redislocation

**Fig. 2:**
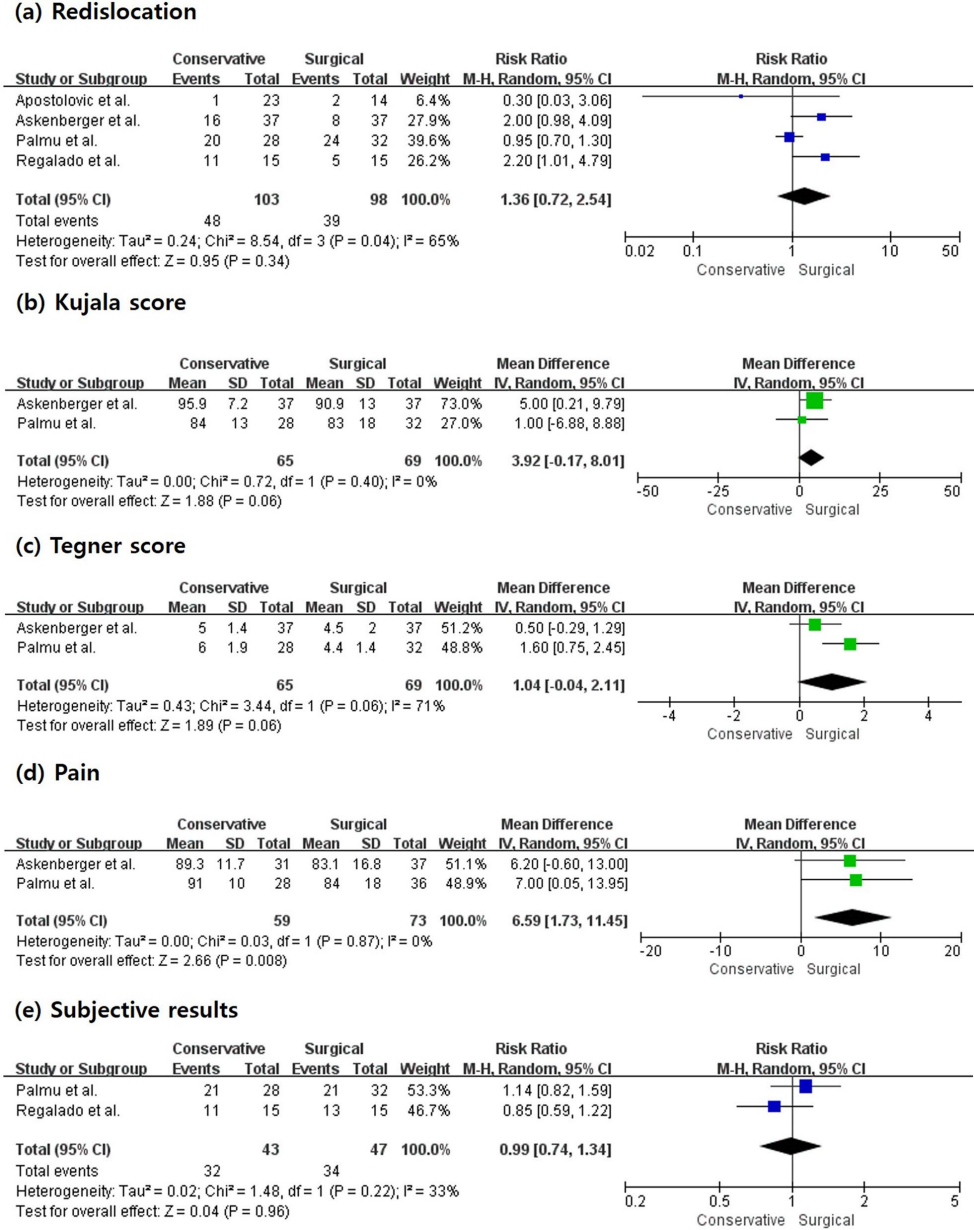
Forest plot showing mean difference or risk ratio in clinical results of conservative versus surgical treatment for patellar dislocation. There were no significant differences in any evaluated outcomes between the two groups (**a** Redislocation, **b** Kujala score, **c** Tegner score, **d** Pain, **e** Subjective results). *RR* risk ratio, *SD* standard deviation, *IV* inverse variance, *CI* confidence interval, *df* degrees of freedom

Four studies reported redislocation in conservative and surgical groups (conservative group/surgical group; 103/98). There were no significant differences in redislocation between conservative and surgical groups (RR 1.36, 95% CI 0.72–2.54, *I*^2^ 65%) (Fig. 2a).

#### Kujala score

Two studies reported Kujala scores for conservative and surgical groups (conservative group/surgical group: 65/69). Remaining studies were excluded due to insufficient data. There were no significant differences in Kujala score between conservative and surgical groups (MD 3.92, 95% CI −0.17 to 8.01, *I*^2^ 0%) (Fig. 2b).

#### Tegner score

Two studies reported Tegner scores for conservative and surgical groups, consisting of a total of 134 subjects (65 subjects in the conservative group and 69 subjects in the surgical group). There was no significant difference in Tegner score between conservative and surgical groups (MD 1.04, 95% CI −0.04 to 2.11, *I*^2^ 71%) (Fig. 2c).

#### Pain

Two studies reported pain in conservative and surgical groups, consisting of a total of 132 subjects (59 subjects in the conservative group and 73 subjects in the surgical group). There were significant differences in pain between conservative and surgical groups (MD 6.59, 95% CI 1.73–11.45, *I*^2^ 0%) (Fig. 2d).

#### Subjective results

Two studies reported subjective results for conservative and surgical groups, consisting of a total of 90 subjects (43 subjects in the conservative group and 47 subjects in the surgical group). There were no significant differences in subjective results between conservative and surgical groups (RR 0.99, 95% CI 0.74–1.34, *I*^2^ 33%) (Fig. 2e).

## Discussion

The most important finding of the present study was that surgical treatment for acute patellar dislocation in children and adolescents was not superior to conservative treatment in terms of clinical outcomes including redislocation, Kujala score, Tegner score, and subjective results. The results indicate that conservative treatment may be a preferable option for primary acute patellar dislocation, especially in terms of pain. This treatment approach is less invasive than surgical treatment and has been found to be equally effective. Therefore, our hypothesis is supported by these findings. Furthermore, these results are the same as those of conservative and surgical treatment in primary patellar dislocation of adults [13]. Until the end of the twentieth century, conservative treatment was frequently used to treat acute patellar dislocation. However, studies have reported a considerable occurrence (up to 44%) of residual patellar instability, anterior knee pain, and redislocation associated with this approach [32]. Due to advances in proper understanding of functional anatomy and biomechanics of the medial patellar stabilizer, primary surgical treatment has been expanded for acute patellar dislocation [33, 34]. Therefore, surgical treatment may be prioritized as the primary treatment option to not only minimize the chance of reoccurrence, but also to optimize functional recovery and facilitate a prompt return to sports activities. Despite the advocacy of these surgical issues, in our study, the clinical results of the two groups did not show significant differences, and the conservative treatment group showed favorable results regarding pain. In addition, since the original study included in the analysis of pain did not show a statistically significant difference between the two groups, additional large-scale studies are needed for more strengthening evidence.

Recent studies on acute patellar dislocation have involved mainly adult patients and demonstrated that both conservative and surgical treatment could be feasible options for treating acute patellar dislocation [35–38]. However, studies on acute patellar dislocation in children and adolescents comparing clinical outcomes after conservative and surgical treatment are rare. According to McManus et al. [39], the incidence of recurrence after an acute patellar dislocation is estimated to be six children out of every ten. Additionally, several other studies have reported a particularly high risk of recurrence in pediatric patients, with some reporting redislocation rates as high as 60% [40, 41]. Despite these high redislocation rates, many authors stated that there was no significant difference in clinical results between the two groups. For instance, Regalado et al. [10] have reported a significantly higher redislocation rate in conservative group compared with operative group. However, they suggest that both treatments are feasible options for treating acute patellar dislocation in adolescents. In the same vein, Askenberger et al. [28] have reported that surgical repair of a MPFL injury in skeletally immature children with a primary traumatic patellar dislocation can significantly reduce the redislocation rate without improving subjective or objective knee function compared with a knee brace without repair. In addition, Palmu et al. [8] have reported that initial surgical repair of medial structures could not improve long-term outcomes with a very high rate of recurrent instability. As such, in many studies, redislocation rates in conservative and surgical groups have been reported differently for each study, but there was no significant difference in the clinical results of the two groups. Furthermore, to establish stronger evidence, it was also possible to confirm a systematic review and meta-analysis study that studied topics similar to the present study. However, compared with our study, one study only analyzed redislocation and lacked analysis of other clinical results [42]. In addition, there were studies that did not include statistical analysis, so there were limitations in obtaining reliable results, and the level of evidence was low because level I and II studies (high quality studies) were omitted from the analysis or retrospective studies were included [9, 43]. Unlike those studies, our study involved only RCTs and prospective studies (level I and II). These included trials showed a low risk of bias, indicating that most studies were of good quality. Thus, this meta-analysis has strong evidence for treating acute patellar dislocation in children and adolescents.

Although our study has several strengths, it also has some limitations. One major limitation is the relatively small number of studies included in the meta-analysis for each topic. Additionally, there were few prospective studies available, which is a significant limitation. Nevertheless, we considered each study result to be of clinical value and therefore included them in our analysis. The strength of this study was its study design: a systematic review and meta-analysis including randomized controlled trials and prospective study on skeletally immature children and adolescents. Second, although not analyzed in our study, several risk factors for recurrent patellar dislocation that might affect clinical outcomes should be considered. Jaquith et al. [44] have demonstrated that trochlear dysplasia, skeletal immaturity, patellar alta, and a history of contralateral patellar dislocation are all significant risk factors for recurrence in patients with first-time patellar dislocation. Thus, various risk factors that might affect recurrent patellar instability following treatment of acute patellar dislocation need to be controlled, including trochlear dysplasia, vastus medialis oblique dysplasia, hyperlaxity, increased patellar tilt, patellar alta, and increased tibial tuberosity to trochlear groove distance [41, 45]. Third, technical factors of surgery that may affect results following surgical treatment need to be controlled. One study performed MPFL repair, and another study performed LRR. There were also studies using two methods combined. In addition, although MPFL reconstruction is widely performed as a treatment for dislocation of the patella, it has not been sufficiently discussed due to the lack of research data related to MPFL reconstruction in children and adolescents. To solve this heterogeneity, a larger, well-controlled prospective study is needed. Controlling for confounding factors in a study is crucial to obtaining accurate results. In an ideal study, all confounding factors would be removed to evaluate the independent factor. Randomization is performed to minimize confounding factors and distribute them equally among study groups [46]. However, in practice, controlling for all confounding factors affecting clinical outcomes is limited. While a meta-analysis can provide powerful conclusions by analyzing studies on the same topic, it is not possible to exclusively include studies with the same methodology. To minimize bias, inclusion criteria recommended by the Cochrane collaboration guideline were strictly adopted when selecting papers for the study. Only randomized and prospective studies (level I and II studies) were included using a random effects model statistically, which helped to minimize the risk of bias. However, it should be noted that the limited number of studies available on each topic is a potential limitation of this study.

## Conclusions

Despite of better outcome in pain with conservative group, the present study revealed that there were no significant differences in clinical outcomes between conservative treatment and surgical treatment in children and adolescents with acute patellar dislocation. Since there were no clear differences in clinical outcomes between the two groups, routine surgical treatment is not advocated for treating acute patellar dislocation in children and adolescents.

## Data Availability

All data generated or analyzed during this study are included in this published article.

## Appendix

Electronic search strategy for each database.

MEDLINE

1. “Medial patellofemoral ligament”[tiab] OR “MPFL”[tiab], *n* = 1233
2. “Recurrent patellar dislocation” [tiab] OR “recurrent patellar instability”[tiab], *n* = 525
3. "Patellar dislocation"[Mesh], *n* = 1425
4. 1 OR 2 OR 3, *n* = 2212
5. Medial reefing[tiab] OR Medial augmentation[tiab] OR Medial plication[tiab] OR Medial imbrication[tiab] OR Quadricepsplasty[tiab] OR Proximal realignment[tiab], *n* = 271
6. Single-Bundle[tiab] OR Double-Bundle[tiab] OR ((Single[tiab] OR Double[tiab]) AND Bundle[tiab]), *n* = 6822
7. 5 OR 6, *n* = 7093
8. 4 AND 7, *n* = 120
9. 8 NOT ("review"[Publication Type] OR "review literature as topic"[MeSH Terms]), *n* = 110

EMBASE

1. 'knee ligament'/de OR 'knee cruciate ligament'/exp, *n* = 27,734
2. "Medial patellofemoral ligament":ab,ti, *n* = 1296
3. “Patellar dislocation”:ab,ti, *n* = 1392
4. 1 OR 2 OR 3, *n* = 29,388
5. Medial reefing:ab,ti OR Medial augmentation:ab,ti OR Medial plication:ab,ti OR Medial imbrication:ab,ti OR Quadricepsplasty:ab,ti OR Proximal realignment:ab,ti, *n* = 477
6. 4 AND 5, *n* = 83
7. 6 NOT ('conference review'/it OR 'review'/it), *n* = 71

COCHRANE

1. “Medial patellofemoral ligament” OR “MPFL”:ti,ab,kw, *n* = 86
2. MeSH descriptor: [Patellar dislocation] explode all trees, *n* = 49
3. 1 OR 2, *n* = 111
4. Medial reefing OR Medial augmentation OR Medial plication OR Medial imbrication OR Quadricepsplasty OR Proximal realignment:ti,ab,kw, *n* = 152
5. Single-Bundle OR Double-Bundle OR ((Single OR Double) AND Bundle):ti,ab,kw, *n* = 806
6. 4 OR 5, *n* = 950
7. 3 AND 6, *n* = 16
8. 7/trials, *n* = 15

## Abbreviations

RCT: Randomized controlled trial
MPFL: Medial patellofemoral ligament
PT: Physical therapy
LRR: Lateral retinacula release
VAS: Visual analogue scale
KOOS: Knee injury and osteoarthritis outcome score
SD: Standard deviation
MD: Mean difference
CI: Confidence interval
RR: Risk ratio
